# Clinicopathologic features and prognostic factors for patients with colorectal cancer who are 75 years and older

**DOI:** 10.18632/oncotarget.20656

**Published:** 2017-09-06

**Authors:** Mingfang Zhao, Hans Liu, Yanqing Tang, Xin Meng, Jun Yu, Qi Wang, Qiao Zhou, Sean X. Leng, Haiyan Zhang

**Affiliations:** ^1^ Department of Medical Oncology, The First Hospital of China Medical University, Shenyang, P.R. China; ^2^ Division of Geriatric Medicine and Gerontology, Department of Medicine, Johns Hopkins University School of Medicine, Baltimore, MD, USA; ^3^ Department of Psychiatry, The First Affiliated Hospital of China Medical University, Shenyang, P.R. China; ^4^ Department of Biochemistry and Molecular Biology, College of Basic Medical Sciences of China Medical University, Shenyang, P.R. China; ^5^ Department of Pathology, Johns Hopkins University School of Medicine, Baltimore, Maryland, USA; ^6^ Department of Geriatrics, The First Hospital of China Medical University, Shenyang, P.R. China

**Keywords:** clinicopathologic features, prognostic factors, colorectal cancer, aged 75 years and over

## Abstract

Colorectal cancer (CRC) is common and can be considered as a disease of older adults. About one half of the cases were diagnosed in patients over 70 years of age. Decision-making about treatment for these older patients can be complicated by age-related physiological changes, impaired functional status, limited social support, and comorbidities. Many trials excluded patients using an upper limit of 75 years of age. Little is known about prognostic factors in patients who are over this age limit. In this study, we conducted an analysis in the Surveillance, Epidemiology and End Results (SEER) database to identify specific clinicopathologic features and prognostic factors for these vulnerable cancer patients (N= 293,616). They were predominantly female and had more stage I and II diseases in comparison to younger patients. On average, these patients had lower 5-year cause-specific mortality than younger patients (41.98% vs. 63.14%, P<0.001). Gender, marital status, ethnicity, Tumor-Node-Metastasis stage, grade, histologic subtype, tumor size, status of surgery and radiotherapy were all independent prognostic factors for these elderly CRC patients. In particular, surgery could improve prognosis for all CRC patients with the exception of those who are older than 94 years old and with stage III disease. The identified clinicopathologic features and prognostic factor will help guide treatment decision-making for this oldest old subset of patients with CRC.

## INTRODUCTION

Colorectal cancer (CRC) is among the leading causes of cancer-related deaths worldwide [1, 2]. The median age of CRC patients was around 69 years old [3]. Decision-making with regards to treatment for older CRC patients may be complicated by certain age-related physiological changes, impaired functional status, limited social support, ability to tolerate treatment toxicity, and presence of comorbidities [4–7]. Many clinical trials have set 75 years as their upper age limit for study enrollment, excluding the oldest old subset [8]. Evidence for the treatment in older adults were derived primarily from individual trial or pooled subgroup analyses [9, 10] [11, 12] and large population-based studies [13, 14]. Because these studies only evaluated the effects of chemotherapy on CRC, it remains unknown whether patients over 75 years of age would benefit from surgery or radiation therapy. Moreover, little is known about clinicopathologic features and prognostic factors specific for this older and frail subset of CRC patients.

To address these questions, we analyzed the clinical data of CRC patients in the Surveillance, Epidemiology, and End Results (SEER) database. Herein, our analysis unveiled clinical-pathological factors of both younger and older CRC patients. We have further examined prognostic factors for the oldest old subset.

## RESULTS

### Patient baseline characteristics

A total of 293,616 patients with colorectal cancer, adenocarcinoma type, were identified from the SEER database. Their median age was 68 years. Among these patients, 100,719 (34.3 %) were 75 years or older and 192,897 (65.7%) were younger than 75. Table 1 shows the basic characteristics of these two group of patients.

**Table 1:** Basic characteristics between younger and older patients

	Age<75 N (%)	Age≥75 N(%)	P value
Gender			
Female	84,989 (44.06)	55,597 (55.2)	
Male	107,908 (55.94)	45,122 (44.8)	<0.001
Age			
(Mean ± SD)	59.78±10.10	81.95±5.00	<0.001
AJCC 6^th^ TNM stage			
I	50,354 (26.10)	27,716 (27.52)	
II	47,324 (24.53)	32,582 (32.35)	
III	55,333 (28.69)	25,532 (25.35)	
IV	39,886 (20.68)	14,889 (14.78)	<0.001
AJCC 6^th^ T stage			
T0	242 (0.13)	117 (0.12)	
T1	37,325 (19.35)	16,801 (16.68)	
T2	25,903 (13.43)	15,722 (15.61)	
T3	94,723 (49.11)	51,414 (51.05)	
T4	26,291 (13.63)	12,755 (12.66)	
TX	8,413 (4.36)	3,910 (3.88)	<0.001
AJCC 6^th^ N stage			
N0	108,192 (56.09)	65,016 (64.55)	
N1	49,003 (25.40)	21,359 (21.21)	
N2	30,394 (15.76)	11,801 (11.72)	
NX	5,308 (2.75)	2,543 (2.52)	<0.001
AJCC 6^th^ M stage			
M0	153,011 (79.32)	85,830 (85.22)	
M1	39,886 (20.68)	14,889 (14.78)	<0.001
Histology			
Adenocarcinoma	175,304 (90.88)	89,796 (89.15)	
Mucinous	15,390 (7.98)	9,935 (9.86)	
Signet ring cell	2,203 (1.14)	988 (0.98)	<0.001
Location			
RSCC	57,291 (29.70)	45,584 (45.26)	
LSCC	76,463 (39.64)	29,534 (29.32)	
Rectal cancer	42,952 (22.27)	14,457 (14.35)	
Others	16,191 (8.39)	11,144 (11.06)	<0.001
Race			
Caucasian	148,882 (77.18)	85,964 (85.35)	
African	25,659 (13.30)	7,845 (7.79)	
American			
Asian	14,675 (7.61)	6,041 (6.00)	
Others	3,681 (1.91)	869 (0.86)	<0.001
Grade			
Well	16,070 (8.33)	8,361 (8.30)	
Moderate	127,468 (66.08)	65,147 (64.68)	
Poorly	29,027 (15.05)	17,625 (17.5)	
Undifferentiated	3,106 (1.61)	1,870 (1.86)	
Unknown	17,226 (8.93)	7,716 (7.66)	<0.001
Surgery			
Yes	173,503 (89.95)	90,026 (89.38)	
No	19,175 (9.94)	10,581 (10.51)	
Unknown	219 (0.11)	112 (0.11)	<0.001
Radiation			
Yes	33,375 (17.30)	7,535 (7.48)	
No	157,700 (81.75)	92,411 (91.75)	
Unknown	1,822 (0.94)	773 (0.77)	<0.001
Tumor size			
Mean±SD (mm)	45.79±35.29	46.15±33.60	<0.001
Diagnosis			
Microscopically confirmed	192,692 (99.89)	100,508 (99.79)	
Not microscopically confirmed	152 (0.08)	189 (0.19)	
Unknown	53 (0.03)	22 (0.02)	<0.001
Married status			
Married	114,575 (59.40)	44,901 (44.58)	
Unmarried	69,453 (36.01)	51,228 (50.86)	
Unknown	8,869 (4.60)	4,590 (4.56)	<0.001
Insurance status			
Insured	126,038 (65.34)	65,862 (65.39)	
Uninsured	6,341 (3.29)	240 (0.24)	
Unknown	60,518 (31.37)	34,617 (34.37)	<0.001

Abbreviation: SD: Standard deviation; AJCC: American Joint Committee on Cancer TNM: Tumor-Node-Metastasis; RSCC: right-sided colon cancer; LSCC: left-sided colon cancer.

In comparison to the young, the older group had a significantly higher proportion of female (55.2% vs. 44.1%, *P* < 0.001). For the stage of Tumor-Node-Metastasis (TNM), the older patients had a higher percentage of stage I and II diseases. Signet-ring cell carcinoma were less common in the older group, while poorly differentiated and undifferentiated adenocarcinoma were more common in older patients. The older patients had more tumors within the right-sided colon, more Caucasians, and more who were unmarried. In terms of treatment for CRC, significantly fewer patients in the older group received surgery or radiotherapy.

### Survival analysis

**Figure 1:**
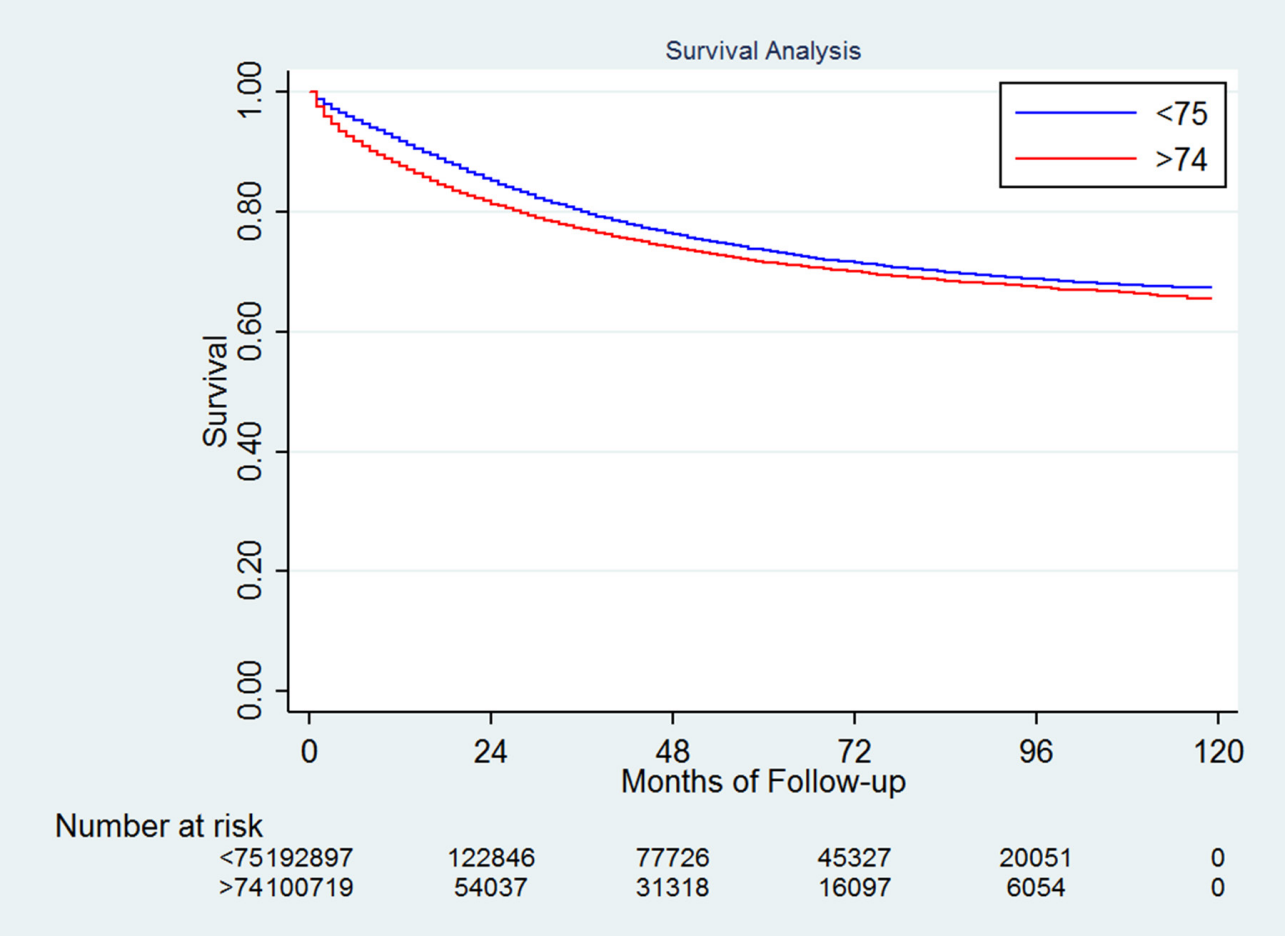
Survival difference between the younger and older patients

The 5-year cause-specific survival (CSS) for the entire study population was 55.71% [95% confidence interval (CI): 55.50%-55.91%]. There were 55,436 deaths (55.04 %) in the older group and 63,247 (32.79%) in the young group. Consistently, the 5-year CSS was significantly higher in the younger patients than in the older patients, 63.14% vs. 41.98%, *P*<0.001 (Figure 1). The 5-year other cause of survival rate was 91.83% and 69.23% for the younger and older patients, respectively, *P*<0.001.

We then analyzed the prognostic factors for patients 75 years old and over (Table 2). Not surprisingly, the stage of TNM was significantly correlated with CSS – the 5-year CSS were 55.78%, 50.66%, 37.44%, and 4.57% for patients with stage I, II, III, and IV, respectively, *P*<0.001. As for the histology subtypes, patients with signet ring cells had a worse 5-year CSS compared to those with mucinous adenocarcinoma or other adenocarcinoma (25.63% vs. 40.91% vs. 42.28%, P<0.001). When we analyzed the 5-year CSS in patients with different tumor grades, we found that the disease-specific survival worsened as the grade progressed from “well” to “un-differentiated” (49.85%, 45.03%, 35.05%, and 33.25% for well-, moderately, poorly, and un-differentiated tumors, respectively, *P*<0.001).

**Table 2:** Survival analysis in older patients

	5yr-CSS	95% CI	*P* value
Gender			
Male	39.34%	38.82%-39.87%	
Female	44.12%	43.64%-44.60%	<0.001
Marry status			
Married	45.62%	45.09%-46.16%	
Unmarried	38.57%	38.08%-39.05%	
Unknown	44.73%	42.99%-46.46%	<0.001
Insurance status			
Insured	41.98%	41.48%-42.47%	
Uninsured	45.83%	37.47%-53.78%	
Unknown	41.94%	41.41%-42.46%	0.5046
Ethnicity			
Caucasian	42.24%	41.85%-42.62%	
African American	34.83%	33.59%-36.06%	
Asian	47.35%	45.84%-48.83%	
Others	44.68%	40.66%-48.62%	<0.001
Site			
LSCC	42.39%	41.74%-43.03%	
RSCC	44.43%	43.90%-44.96%	
Rectum	36.30%	35.38%-37.21%	<0.001
AJCC 6^th^ TNM stage			
I	55.78%	55.10%-56.46%	
II	50.66%	50.02%-51.30%	
III	37.44%	36.74%-38.14%	
IV	4.57%	4.18%-5.00%	<0.001
Grade			
Well differentiated	49.85%	48.62%-51.07%	
Moderately differentiated	45.03%	44.58%-45.47%	
Poorly differentiated	35.05%	34.25%-35.84%	
Undifferentiated	33.25%	30.55%-35.97%	
Unknown	25.53%	24.42%-26.65%	<0.001
Histology			
Other adenocarcinoma	42.28%	41.90%-42.66%	
Mucinous adenocarcinoma	40.91%	39.80%-42.00%	
Signet ring cell	25.63%	22.55%-28.80%	<0.001
Size			
<=40mm	48.52%	47.97%-49.07%	
>40mm	37.05%	36.59%-37.50%	<0.001
Surgery			
Yes	73.0%	72.7%-73.2%	
No	9.3%	8.6%-10.1%	
Unknown	26.8%	19.8%-34.3%	<0.001
Radiation			
No	46.0%	44.0%-48.0%	
Yes	69.8%	69.5%-70.1%	
Unknown	62.5%	59.1%-65.8%	<0.001

Abbreviation: CSS: Cause specific survival; CI: Confidence interval; AJCC: American Joint Committee on Cancer TNM: Tumor-Node-Metastasis; RSCC: right-sided colon cancer; LSCC: left-sided colon cancer.

Among the older CRC patients, about 89% underwent surgery for CRC and 7.48% received radiation therapy (78.3% of patients who received radiotherapy were rectal adenocarcinoma patients). The older patients who have underwent surgery had a significantly better prognosis in comparison to those who have not.

Moreover, females had a better prognosis compared to the males. The other factors that affect prognosis includes marital status, ethnicity, location of primary tumor, and tumor size. In contrast, whether the patients were covered by insurance or not had no impact on the disease-specific survival.

### Multivariate analysis

Variables showing a trend for association with survival (*P* < 0.05) were selected in the cox proportional hazards model. Gender, married status, ethnicity, location, TNM stage, histologic subtypes, grade, tumor size, radiation as well as surgery were all independent prognostic factors in the multivariable analysis (Table 3).

**Table 3:** Multivariate analysis of survival in older patients

	Hazard ratio	Standard error	*P* value	95% confidence interval
Sex				
Male	Reference			
Female	0.89	0.005	<0.001	0.86-0.91
Ethnicity				
Caucasian	Reference			
Asian	0.83	0.011	<0.001	0.81-0.86
African-American	1.02	0.009	0.012	1.01-1.04
Others	0.87	0.02	<0.001	0.83-0.92
Married status				
Married	Reference			
Unmarried	1.44	0.009	<0.001	1.42-1.46
Unknown	1.12	0.018	<0.001	1.08-1.15
Site				
RSCC	Reference			
LSCC	0.82	0.006	<0.001	0.81-0.83
Rectum	0.86	0.009	<0.001	0.84-0.87
AJCC 6^th^ TNM stage				
I	Reference			
II	1.29	0.013	<0.001	1.27-1.32
III	1.72	0.017	<0.001	1.69-1.76
IV	5.23	0.054	<0.001	5.12-5.33
Histology subtype				
Adenocarcinoma	Reference			
Mucinous adenocarcinoma	1.13	0.012	<0.001	1.11-1.15
Signet ring cell	1.42	0.034	<0.001	1.36-1.49
Grade				
Well differentiated	Reference			
Moderate differentiated	1.09	0.014	<0.001	1.07-1.12
Poorly differentiated	1.46	0.021	<0.001	1.42-1.50
Undifferentiated	1.61	0.041	<0.001	1.54-1.69
Unknown	1.16	0.019	<0.001	1.13-1.20
Surgery				
No	Reference			
Yes	0.38	0.004	<0.001	0.37-0.39
Unknown	0.71	0.028	<0.001	0.66-0.77
Radiation				
Yes	Reference			
No	1.25	0.014	<0.001	1.22-1.28
Unknown	1.30	0.042	<0.001	1.22-1.39
Size				
<=40mm	Reference			
>40mm	1.09	0.007	<0.001	1.08-1.10

Abbreviation: RSCC: right-sided colon cancer; LSCC: left-sided colon cancer; AJCC: American Joint Committee on Cancer TNM: Tumor-Node-Metastasis.

### Who benefited from surgery?

To better understand the role and benefit of surgery in treatment of patients with CRC who were over 75 years of age patients, we analyzed these patients according to their disease stage and age. We first stratified the patients into 5 age groups – 75-79, 80-84, 85-89, 90-94, and >94 years old. Surgery did not bring survival benefit to the patients who were older than 94 years old and with stage III diseases. As for the rest of the patients, those who received surgery for CRC had a significantly improved 5-year CSS as compared to those did not undergo such surgery (Table 4). Moreover, for the patients with the same stage of TNM, the prognosis for CRC worsened with increasing age.

**Table 4:** The 5-year CSS for older patients with or without surgery

	Surgery	No surgery	*P value*
Stage I			
75-79 years old	70.00% (68.95%-71.01%)	28.38% (23.92%-32.98%)	<0.001
80-84 years old	58.98% (57.75%-60.19%)	15.99% (12.93%-19.35%)	<0.001
85-89 years old	47.41% (45.73%-49.06%)	9.91% (7.35%-12.92%)	<0.001
90-94 years old	36.83% (33.76%-39.90%)	1.80% (0.59%-4.32%)	<0.001
>94 years old	22.91% (16.53%-29.93%)	2.06% (0.2%-8.85%)	<0.001
Stage II			
75-79 years old	61.73% (60.66%-62.78%)	17.31% (11.83%-23.67%)	<0.001
80-84 years old	54.07% (52.95%-55.18%)	13.18% (9.18%-17.92%)	<0.001
85-89 years old	42.97% (41.57%-44.36%)	8.35% (4.47%-13.75%)	<0.001
90-94 years old	28.66% (26.46%-30.90%)	8.45% (3.71%-15.67%)	<0.001
>94 years old	21.07% (16.64%-25.87%)	0	0.0006
Stage III			
75-79 years old	47.81% (46.64%-48.98%)	9.15% (4.25%-16.35%)	<0.001
80-84 years old	37.81% (36.60%-39.02%)	9.56% (4.81%-16.26%)	<0.001
85-89 years old	27.94% (26.46%-29.44%)	5.00% (1.49%-11.86%)	<0.001
90-94 years old	18.75% (16.54%-21.07%)	8.62% (2.75%-18.81%)	<0.001
>94 years old	11.99% (7.99%-16.87%)	15.41% (1.12%-45.95%)	0.2678

Abbreviation: CSS: cause specific survival.

## DISCUSSION

Colorectal cancer is a highly prevalent malignancy in older adults worldwide [15–17]. With rapid growth of older adults in numbers, particular the oldest old subset aged 75 years and over, the number of older patients with CRC and those who seek treatment will increase rapidly. However, many clinical trials set an upper age limit of 75 years for study enrollment. Even in trials with no such age limit, they only enrolled a small number of patients with advanced age. These limited number of patients are unlikely representative of the general geriatric population with CRC. To address this important issue, we took the advantage of the existing data in the SEER, a composite population-based cancer registry covering several discrete geographic regions. We found that about one third of the CRC patients were of age 75 or over. We then further analyzed their clinicopathologic features as well as prognostic factors.

Firstly, we identified a number of unique clinicopathologic features in this oldest old subset. In comparison to the young group, these older patients were more likely single or widowed. They had larger and more poorly differentiated tumor and their tumor was more likely located in the ascending colon. On the other hand, these older patients had less metastasis of primary tumor and less signet ring cell carcinoma. Nonetheless, the 5-year CSS was lower in the older patients (*P* < 0.05). Two previous studies reported age as an independent negative prognostic factor in stage I-IV colon cancers [3, 18] in which “older patients” were defined differently; one study used median age (69 years) as the cutoff value [3] while the other used 40 years of age as the cutoff value [18]. This study, for the first time, reports 5-year CSS in patients with CRC who were 75 years old and over, which was nearly 42%.

Much remains to be learned about the prognostic factors for this oldest subset of elderly patients with CRC. In this study, we identified that gender, marital status, ethnicity, the stage of TNM, grade, tumor histologic subtype, tumor size, surgical intervention and radiotherapy were all independent prognostic factors for these older patients. These prognostic factors were not much different from other age groups [3, 14, 19].

As this oldest subset of elderly patients are more often challenged by age-related physiological changes, impaired functional status, limited social support, decreased ability to tolerate treatment toxicity, and presence of comorbidities, it is unclear if they would benefit from cancer treatments similar to the younger patients. Here, we report that surgical intervention provided survival benefit for most patients in this age group except for those who were both over 94 years of age and had stage III disease. Radiation therapy also offered survival benefits for patients with rectal cancer in this age group. We focused our analysis on patients with rectal cancer because they accounted for over 80% of those receiving radiation therapy. Consistent with these results, Pérez Domínguez L et al. found that age did not affect the prognosis after colon cancer resection but was associated with more postoperative morbidity and mortality [22].

This study has limitations. For example, information about chemotherapy was not available in the SEER database. Thus, potential survival benefit of chemotherapy for the oldest old subset of patients with CRC could not be determined in this study, However, subgroup and pooled analyses o phase III clinical trials suggested that the relatively fit older patients with CRC who met the traditional clinical trial inclusion criteria were likely to experience survival benefits from combination Oxaliplatin as the first line therapy similar to the younger patients [20, 21], supporting the hypothesis that the oldest old subset patient would also benefit from first line chemotherapy. Another limitation is that no data were available in the SEER database on comorbidities which are prevalent in the oldest old subset patients and known to influence prognosis and treatment decision-making for these patients. Finally, given that this is a retrospective cohort study, there is potential bias of nonrandomized data between the focused oldest old subset and younger patients with CRC. Despite these limitations, our study has identified specific clinicopathologic features and prognostic factors of the elderly patients with CRC who are 75 years and older. These data will help guide treatment decision-making for this specific and rapid growing group of elderly patients with CRC. In summary, our findings, for the first time showed that the oldest old subset of elderly CRC patients not only had a relatively poor prognosis compared to their younger counterparts but also could potentially benefit from surgery and radiotherapy.

## MATERIALS AND METHODS

### Database

The Surveillance, Epidemiology, and End Results (SEER) database is the largest publicly available cancer dataset. It is a composite population-based cancer registry. The SEER research data include cancer incidence and prevalence as well as patient demographics as tabulated by age, sex, race/ethnicity, and year of cancer diagnosis. The specific dataset used for this study was the SEER Program (http://www.seer.cancer.gov) Research Data (1973-2013).

### Outcome variables

The anatomic sites of the left colon, right colon, and rectum were categorized according to the International Classification of Diseases for Oncology, third edition (ICD-0-3) topography codes. Right-sided colon cancers were identified by using the following SEER cancer site codes: cecum (ICD-0-3 code C18.0), ascending colon (Code C18.2), hepatic flexure (Code C18.3), and transverse colon (Code C18.4); left-sided colon cancers were identified by using the following codes: splenic flexure (Code C18.5), descending colon (code C18.6), sigmoid colon (code C18.7), and rectosigmoid (code C19.9); rectal cancer was identified by using the code C20.9.

In this study, only adenocarcinoma patients were enrolled (SEER histology codes: signet ring cell, 8490; mucinous adenocarcinoma, 8480 and 8481; other adenocarcinoma: 8140 to 8147, 8210 to 8211, 8220 to 8221, and 8260 to 8263).

For the insurance status, individuals in the groups “Any Medicaid”, “Insured” and “Insured/No specifics” were clustered together as “Insured group”.

### Patient population

The study population was derived from the SEER cancer registry. Individuals identified as colorectal adenocarcinoma patients from 2004 to 2013, inclusive, were eligible for our study. Patients were excluded if they (1) had more than one primary cancer and the CRC was not the first to appear, and/or (2) had unknown cause(s) of death or unknown survival months. All the patients included in the study had active follow up and a survival of over 1 month.

Age, sex, tumor stage according to the American Joint Committee on Cancer (AJCC) Cancer Staging Manual (6^th^ edition, 2004), tumor histological subtype, tumor grade, insurance status, marital status, time of disease diagnosis, survival time, and CSS were extracted from the SEER database.

### Statistical methods

The patients’ demographic characteristics and tumor characteristics are summarized using descriptive statistics (Table or Figure). Comparisons of specific categorical variables between the older and younger patients were performed using the Chi squared test, and continuous variables were compared using the Student’s t-test. The primary endpoint of this study was the 5-year CSS, as calculated from the date of diagnosis to the date of cancer-specific death. Deaths attributed to CRC were treated as events, and deaths from other causes were treated as censored observations. Survival function estimation and comparison between older and younger patients were performed using Kaplan–Meier estimates and log-rank test, respectively. The multivariate Cox proportional hazard model was used to evaluate the hazard ratio (HR) and the 95 % CI for all the known prognostic factors for CRC. All statistical analyses were performed using the Intercooled Stata 13.0 (Stata Corporation, College Station, TX). Statistical significance was set at two-sided *P* < 0.05.

## Abbreviations

AJCC: American Joint Committee on Cancer
CI: confidence interval
CRC: Colorectal cancer
CSS: cause-specific survival
HR: hazard ratio
ICD-O: International Classification of Diseases for Oncology
SEER: Surveillance, Epidemiology, and End Results
TNM: Tumor-Node-Metastasis
